# Reentrant condensation of a multicomponent cola/milk system induced by polyphosphate

**DOI:** 10.1016/j.fochx.2024.101165

**Published:** 2024-01-28

**Authors:** Tomohiro Furuki, Tomohiro Nobeyama, Shunji Suetaka, Ryokei Matsui, Tatsuhiko Fukuoka, Munehito Arai, Kentaro Shiraki

**Affiliations:** a Faculty of Pure and Applied Sciences, University of Tsukuba, 1-1-1 Tennodai, Tsukuba, Ibaraki 305-8573, Japan; b Department of Integrated Sciences, College of Arts and Sciences, The University of Tokyo, 3-8-1 Komaba, Meguro, Tokyo 153-8902, Japan; c Ishikawa Prefectural Nanao High School, E-1-1 Nishi-fujihashi, Nanao, Ishikawa 926-0817, Japan; d Department of Life Sciences, Graduate School of Arts and Sciences, The University of Tokyo, 3-8-1 Komaba, Meguro, Tokyo 153-8902, Japan; e Department of Physics, Graduate School of Science, The University of Tokyo, 3-8-1 Komaba, Meguro, Tokyo 153-8902, Japan

**Keywords:** Reentrant condensation, Cola, Milk, Polyphosphate, Casein

## Abstract

Reentrant condensation (RC) is a protein behavior in which the protein solution shifts between the one- and two-phase state more than twice by increasing a single parameter. Although RC would be a candidate mechanism for the physicochemical design of food additives, no realistic model has been established under diverse contaminants like food materials. Here, we found that a mixture of cola and milk yielded RC. At pH 3.2–3.6, cola induced milk condensation at 30–40%, while lower or higher concentrations of cola did not. Furthermore, we reduced this cola/milk system to two pure components, casein in milk and polyphosphate (polyP) in cola, and investigated the characteristics of casein concentration and zeta potential. This was the first experimental demonstration of RC occurrence in a multicomponent system. The well-characterized cola/milk system would explore both the universal nature of proteins and the industrial application of RC.

## Highlights

• A mixture of multicomponent cola and milk yielded reentrant condensation (RC).
• Casein in milk and polyphosphate (polyP) in cola also exhibited reentrant behavior.
• The critical points of RC depended on the length of the polyP chain.
• Electrostatic interaction between casein and polyP induces RC in cola/milk system.
• Introducing the concept of RC could enhance the strategy for designing food additive.

## 1 Introduction

Proteins have diverse states in solution, which regulate their behaviors. The states of proteins in solution are generally classified into one-phase or multi-phase ones. In the one-phase state, protein molecules are homogeneously dispersed, whereas in the multi-phase state, protein molecules are heterogeneously dispersed in at least two phases (typically coacervation (De Kruif, Weinbreck, & De Vries, 2004), aggregation (Stefani & Dobson, 2003), or liquid–liquid phase separation (LLPS) (Shin & Brangwynne, 2017); (Banani, Lee, Hyman, & Rosen, 2017)).

**Fig. 1:**
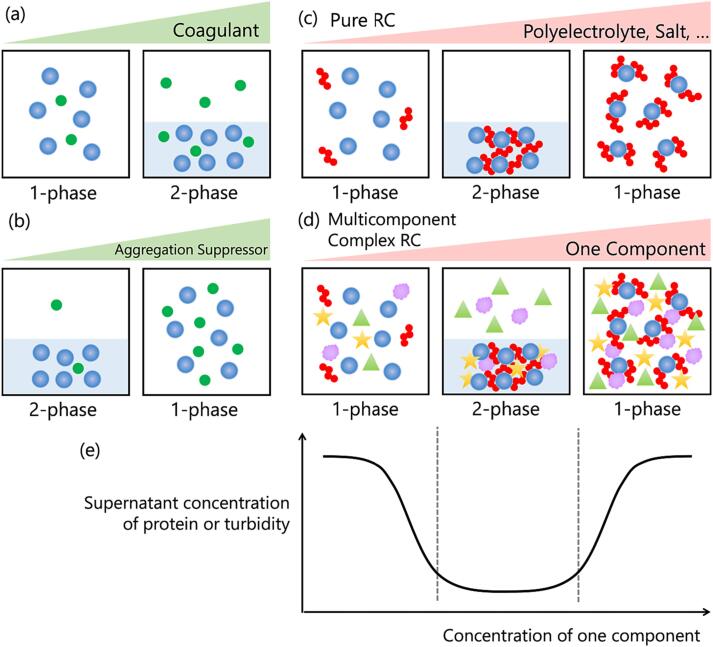
Schematic models of the various phase behaviors of protein solutions. (a) Coagulants (green) induce the condensation of proteins (blue). (b) Aggregation suppressors (green) inhibit the condensation of proteins (blue). (c) Reentrant condensation (RC) in a two-component pure system consisting of a protein (blue) and a polyelectrolyte or salt (red). (d) RC in a multicomponent complex system consisting of a protein (blue), a polyelectrolyte or salt (red), and other components (other colors). In (c) and (d), an increase in the concentration of one component (e.g., polyelectrolyte) induces RC. (e) Supernatant concentration of protein or turbidity depict RC behavior of a system. (For interpretation of the references to color in this figure legend, the reader is referred to the web version of this article.)

The phase behavior of proteins is sensitively affected by their coexisting components, with certain substances able to change this phase behavior. Coagulants are additives that change the protein solution from the one-phase to the two-phase state (Fig. 1a). For example, CaSO_4_ turns soy milk into tofu (soybean curd) (Kohyama, Sano, & Doi, 1995). Similarly, adding polyglutamic acid to an antibody solution induces the separation of the solution into two phases by forming a stable protein–polyelectrolyte complex (PPC) (Kurinomaru & Shiraki, 2017). In turn, aggregation suppressors are additives that prevent the separation of a protein solution into two phases, thus keeping the proteins dispersed in one phase (Fig. 1b); e.g., 0.5 M NaSCN completely prevents the thermal aggregation of hen egg-white proteins and maintains them in one phase, even after heat treatment at 90°C for 30 min (Iwashita, Inoue, Handa, & Shiraki, 2015). In addition, according to their concentration, some additives can both promote and inhibit condensation; e.g., arginine can both prevent antibody aggregation and promote the formation of soluble aggregates (Yoshizawa, Arakawa, & Shiraki, 2017).

Reentrant condensation (RC) is a protein behavior in which the protein solution shifts between the one- and two-phase state more than twice with the increase of a single parameter (Fig. 1c). Recently, RC has been widely observed in proteins and polypeptides (Zhang et al., 2010); (Roosen-Runge, Heck, Zhang, Kohlbacher, & Schreiber, 2013); (Kubíčková et al., 2012); (Banerjee, Milin, Moosa, Onuchic, & Deniz, 2017); (Krainer et al., 2021). Trivalent cations, such as Y^3+^, La^3+^, Fe^3+^, and Al^3+^, induce the RC of globular proteins, such as bovine serum albumin (BSA), human serum albumin, and ovalbumin, because ions with a high charge density can easily induce attractive interactions between ions and proteins (Zhang et al., 2010). The mechanism underlying RC in protein solutions is as follows. Proteins are negatively charged at pH values above their isoelectric point (p*I*). Oppositely charged cations crosslink proteins and cancel out the charges of proteins, thus reducing the repulsion between proteins and polyelectrolytes, ultimately inducing condensation. Further addition of cations reverses the charges on the protein surface, resulting in repulsive forces and the return of the system to the one-phase state (Zhang et al., 2010). The occurrence of such charge inversion is supported by molecular dynamics simulations in a tetraaspartate/trivalent cation system (Kubíčková et al., 2012).

In pure system, small amount of anionic polyphosphate (polyP) induces aggregation of lysozyme through crosslinking, while large amount of polyP stabilizes lysozyme by charge inversion associated with RC (Lenton et al., 2021). Such characteristic of RC would be a new method for the physicochemical design of food additives.

However, the study of protein RC in realistic conditions like food materials remains difficult. Although the phase behavior of proteins is sensitively affected by contaminants, the previous model systems of RC have used very pure systems in the field of physical chemistry. Further study of RC requires a good model of RC under multicontaminant conditions (Fig. 1d).

In the present study, we found that a mixture of cola and milk yielded RC. This RC was reduced by the interaction of two components, casein in milk, and polyP in cola. In cola, polyP is used as a food additive to refine carbon dioxide bubbles and maintain them in an even state after 27 days of storage (Ido & Komine, 2014). In turn, during cheese production, polyP promotes casein aggregation at a neutral pH (Guo, Campbell, Chen, Lenhoff, & Velev, 2003), whereas an excess of polyP suppresses casein aggregation at a neutral pH by imparting a large repulsive negative charge on casein (Mizuno & Lucey, 2007). It is well known in popular science that mixing cola and milk induces condensation (HikakinTV, 2014); however, this has not been regarded as being part of the RC phenomenon. We observed RC in the presence of 20%–60% cola and 1% milk; moreover, a similar RC was detected in a reconstructed pure system containing 0.01–2 mM tetraphosphate (tretaP) and 0.5 mg/ml casein. Supernatant concentration of casein or turbidity were measured to depict RC behavior (Fig. 1e). The mechanism underlying RC consisted in the charge inversion of casein after the addition of polyP. This was the first experimental demonstration of RC in a multicomponent contaminant solution.

## 2 Materials and methods

### 2.1 Materials

Sodium caseinate (casein) was purchased from Wako Pure Chemical Industries (Tokyo, Japan). Sodium tetraphosphate (tetraP), triphosphate (triP), and diphosphate (diP) were purchased from Nacalai Tesque (Kyoto, Japan). NaCl was purchased from Kanto Chemical (Tokyo, Japan). Pepsi Cola was purchased from Suntory (Osaka, Japan). Milk was purchased from Meiji (Tokyo, Japan). Casein and each polyP compound were dissolved separately in Milli-Q water. Cola was used after the removal of carbon dioxide bubbles by sonication. Stock solutions of casein, tetraP, triP, diP, and NaCl were prepared by dissolving each reagent in Milli-Q water (Merck Millipore, Burlington, MA, USA). The concentrations of the stock solutions are as follows: casein (25 mg/ml), tetraP, triP, diP (100 mM), and NaCl (4 M). Sample solutions were prepared by mixing the solutions at the desired concentrations. The pH of each sample was adjusted by adding HCl or phosphoric acid and NaOH (up to 10 mM).

### 2.2 Turbidity measurement

Turbidity was analyzed to assess the color of cola and the turbidity of milk. Sample solutions were prepared by mixing the stock solutions and centrifuged (18,800 × *g*, 20 min, 20°C). Subsequently, the turbidity of the supernatant was determined by measuring the absorption at 400 nm using a NanoDrop ND-1000 instrument (NanoDrop Technologies, Delaware, US) at 25°C.

### 2.3 Casein precipitation analysis

Casein precipitation was analyzed to determine the concentration of casein in the supernatant of the sample solutions. Sample solutions were prepared and centrifuged (18,800 × *g*, 20 min, 20°C). Subsequently, the concentration of casein in the supernatant was determined using the Bradford (Bradford, 1976) or Lowry (Lowry, Rosebrough, Farr, & Randall, 1951) methods. The Bradford protein assay kit was purchased from Takara Bio (Shiga, Japan). Aliquots (4 µl) of supernatant samples were placed in 96-well clear flat-bottom ultra-low attachment microplates (Corning, New York, US), to which 200 µl of Bradford dye reagent was added. After a 30-min incubation, the absorption of each sample at 595 nm was measured using a microplate reader (Infinite 200 PRO, Tecan Japan, Kanagawa, Japan) at 25°C. The measurements were performed in triplicate. The determination of the concentration of casein using the Lowry method was performed with a DC^TM^ protein assay kit purchased from Bio-Rad Laboratories (California, US). Aliquots (5 µl) of supernatant samples were placed in 96-well microplates, to which 25 µl of the DC protein assay reagent A and 200 µl of reagent B were added. After a 30-min incubation, the absorption of each sample at 750 nm was measured using a microplate reader at 25°C. The measurements were performed in triplicate. For each method, a calibration curve was generated using pure casein solutions (0, 0.125, 0.25, 0.5 mg/ml) and was used to determine the concentration of casein.

### 2.4 Zeta potential measurement

The zeta potential was measured on a Zetasizer Nano ZS using DTS1070 cells (Malvern Instruments, Malvern, UK) at 25°C. Samples containing 0.5 mg/ml casein and 0.01–2 mM tetraP were centrifuged (18,800 × *g*, 20 min, 20°C), and the supernatants were collected on the day before the measurements of the zeta potential were performed. The number of runs was 10 at minimum and 30 at maximum.

### 2.5 Generation of phase diagrams of casein in polyP solutions

Phase diagrams of casein in polyP solutions were generated using precipitation measurements. The solutions were mixed and centrifuged (15,000 rpm, 30 min, 4°C). Subsequently, the absorption of the sample supernatants at 280 nm was measured on a UV-1800 spectrophotometer (Shimadzu, Kyoto, Japan) at 25°C. A calibration curve was obtained using pure casein solutions and was used to determine the concentration of casein. Casein was considered to be “condensed” when more than 90% of the protein was precipitated, whereas casein was considered to be “soluble” when less than 90% of the protein was precipitated.

## 3 Results

### 3.1 Reentrant condensation of cola/milk

**Fig. 2:**
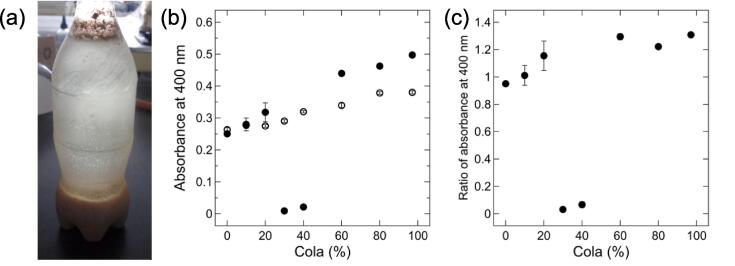
RC in the cola/milk system. (a) Representative photograph of the cloudy white milk precipitates that formed within the dark-brown-colored cola, resulting in a clear supernatant. Movies of the cola/milk mixture can be found on YouTube. (b) Supernatant turbidity of a mixture of 1% milk and different concentrations of cola at pH 3.2–3.6 (closed circles) and pH 7.0–7.4 (open circles). (c) Ratio of the turbidity of the supernatant in the cola/milk system at pH 3.2–3.6 to that at pH 7.0–7.4. The averages and standard deviations of triplicate measurements are shown.

To understand the phase behavior of a cola/milk mixture (Fig. 2a), we investigated dependence of the supernatant turbidity on the concentration of the cola. Solutions at pH 3.2–3.6 and 7.0–7.4 were prepared using a constant concentration of milk (1%) and different concentrations of cola (0%–97%). Fig. 2b depicts the supernatant turbidity of these solutions. At pH 3.2–3.6, the supernatant turbidity decreased to around 0 in the presence of 30%–40% cola, whereas the turbidity was 0.25–0.50 at other cola concentrations. In contrast, at pH 7.0–7.4, the supernatant turbidity was 0.26–0.38 at all cola concentrations tested here. Fig. 2c reports the ratio of the supernatant turbidity at pH 3.2–3.6 to that at pH 7.0–7.4; in the presence of 30%–40% cola, the ratio was below 0.07, whereas it was above 0.95 at other cola concentrations. Interestingly, at pH 3.2–3.6, the turbidity of the supernatant first increased, then decreased, and finally increased again as the concentration of the cola increased. These data indicated that RC occurred in the cola/milk system.

### 3.2 Reentrant condensation of cola/casein

**Fig. 3:**
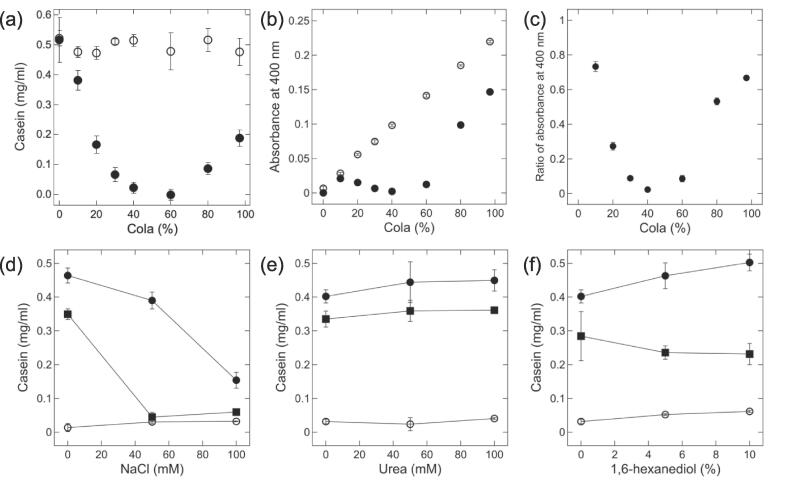
RC in the cola/casein mixture. (a) Casein concentration in the supernatant of a mixture comprising 0.5 mg/ml casein and different concentrations of cola at pH 3.2–3.6 (closed circles) and pH 7.0–7.4 (open circles). (b) Supernatant turbidity of mixtures containing 0.5 mg/ml casein and different concentrations of cola at pH 3.2–3.6 (closed circles) and pH 7.0–7.4 (open circles). (c) Ratio of the supernatant turbidity at pH 3.2–3.6 to that at pH 7.0–7.4 in the cola/casein mixtures. (d-f) Casein concentration in the supernatant of a mixture comprising 0.5 mg/ml casein and different concentrations of NaCl (d), urea (e), and 1,6-hexanediol (f). Closed circles indicate 0% cola, open circles indicate 40% cola, and closed squares indicate 94.5% (d, e) and 80% (f) cola. The averages and standard deviations of triplicate measurements are shown.

Next, casein was selected among the milk components because it is the major milk protein. To understand the phase behavior of the cola/casein mixture, we investigated the effects of the cola concentration on the concentration of casein in the supernatant and on the turbidity of the supernatant. Solutions were prepared using a constant concentration of casein (0.5 mg/ml) and varying concentrations of cola (0%–97%) at pH 3.2–3.6 and 7.0–7.4. Fig. 3a depicts the casein concentration in the supernatant of the cola/casein mixtures containing different concentrations of cola. As the cola concentration increased from 0% to 60%, the concentration of casein in the supernatant decreased from 0.52 to 0 mg/ml. In turn, as the cola concentration increased from 60% to 97%, the concentration of casein in the supernatant increased from 0 to 0.19 mg/ml. Fig. 3b and 3c reports the supernatant turbidity of cola/casein mixtures containing different concentrations of cola. The supernatant turbidity was low in the presence of 30%–60% cola at pH 3.2–3.6, and the ratio of the supernatant turbidity at pH 3.2–3.6 to that at pH 7.0–7.4 decreased to values below 0.1. As observed for the cola/milk system (Fig. 2), the concentration of casein in the supernatant first decreased, and then increased as the cola concentration increased (Fig. 3a). As shown in Fig. 3d, the supernatant concentration of casein in the presence of 0 and 94.5% cola decreased as the concentration of NaCl increased to 100 mM, while casein was still stable in the presence of urea (Fig. 3e), and 1,6-hexanediol (Fig. 3f). These data indicated that RC occurred in the cola/casein system, in which milk is reduced to its major component, casein.

### 3.3 Reentrant condensation of polyP/casein

**Fig. 4:**
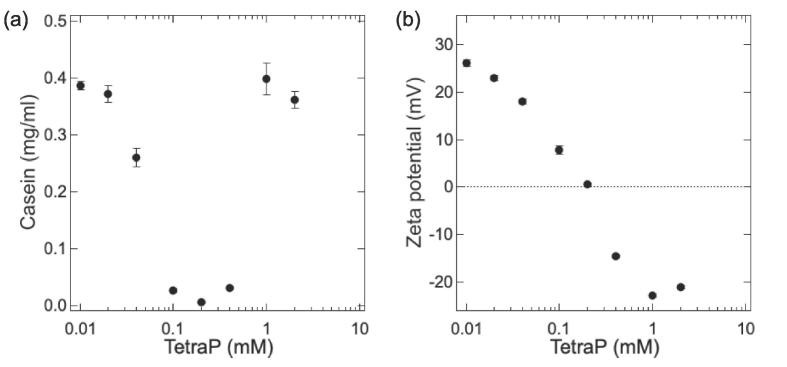
RC and charge inversion of the tetraP/casein mixed solution. Casein concentration (a) and zeta potential (b) in the supernatant of mixed solutions containing 0.5 mg/ml casein and 0.01–2 mM tetraP at pH 3.2–3.6. The averages and standard deviations of triplicate measurements are shown.

Subsequently, polyP was selected as a component of cola, in which it is used as a food additive. To understand the phase behavior of a tetraP/casein mixed solution, we investigated the effects of the concentration of tetraP on the casein concentration and zeta potential in the supernatant of tetraP/casein mixed solutions. Solutions were prepared using a constant concentration of casein (0.5 mg/ml) and varying concentrations of tetraP (0.01–2 mM) at pH 3.2–3.6. Fig. 4a depicts the concentration of casein in the supernatant of tetraP/casein mixed solutions containing different concentrations of tetraP. The concentration of casein was determined using the Lowry method. As the tetraP concentration increased from 0.01 to 0.2 mM, the concentration of casein in the supernatant decreased from 0.39 to 0.01 mg/ml. In turn, as the tetraP concentration increased from 0.2 to 1 mM, the concentration of casein in the supernatant increased from 0.01 to 0.40 mg/ml. Fig. 4b reports the supernatant zeta potential of tetraP/casein mixed solutions containing different concentrations of tetraP. As the tetraP concentration increased from 0.01 to 1 mM, the zeta potential of the supernatant decreased monotonically from 26 to −23 mV, indicating a charge inversion. As observed in the cola/milk (Fig. 2) and cola/casein (Fig. 3) mixtures, the concentration of casein in the supernatant first decreased, and then increased with the increase in the concentration of tetraP (Fig. 4a). These data indicated the occurrence of an RC phenomenon associated with the charge inversion of the tetraP/casein complex.

### 3.4 Phase diagram of casein condensation with different chain length of polyP

**Fig. 5:**
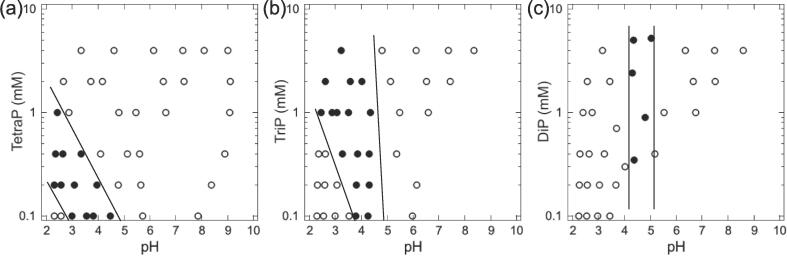
Phase diagram of casein condensation in the presence of (a) tetraP, (b) triP, and (c) diP. The solutions contained 0.5 mg/ml casein and 0.1–4 mM polyP at pH 2–10. Closed circles, condensed state; open circles, soluble state.

To clarify the effects of the length of the polyP chain on the phase behavior of the polyP/casein mixed solutions, we investigated the effect of the concentration of tetraP, triP, or diP at different pH values on the state (condensed or soluble) of the polyP/casein mixed solutions. Solutions containing a constant concentration of casein (0.5 mg/ml) and varying concentrations of tetraP, triP, or diP (0.1–4 mM) were prepared, and the pH was adjusted by adding phosphoric acid. Fig. 5a depicts the phase diagram of casein in the presence of tetraP at different pH values. In the presence of 0.1 mM tetraP, casein condensed at pH 3.0–4.5, but not at pH values below or above this range. In turn, in the presence of 0.2–1.0 mM tetraP, casein was prone to condense at lower pH values. Conversely, casein did not condense at pH values above its p*I* (4.6), regardless of the tetraP concentration tested. Interestingly, the condensation of casein at its p*I* in the presence of 0.1 mM tetraP was suppressed by the addition of tetraP at a concentration of 0.2 mM or higher. These data indicated that tetraP shifts the pH range at which casein condensation occurs toward more acidic values. Fig. 5b provides a diagram of the condensed and soluble states of casein in the presence of triP at different pH values. In the presence of 0.1 mM triP, casein condensed at pH values of 3.8–4.3, which was a narrower range than that observed in the presence of tetraP. The pH range at which casein condensation occurred broadened toward lower values as the triP concentration increased (above 0.1 mM). In turn, casein did not condense at pH values above its p*I* at all triP concentrations tested. Furthermore, we constructed a phase diagram of casein condensation in the presence of diP (Fig. 5c). At all diP concentrations tested, casein condensed at pH values of 4.3–5.0, which were close to the p*I* of casein. Unexpectedly, diP at the concentrations tested did not affect the pH values at which casein condensation occurred, indicating that the negative charge of diP was not sufficient to modify the pH range that triggered casein condensation.

## 4 Discussion

Here, we generated a multicomponent complex system that yielded an RC behavior using cola/milk. At pH 3.2–3.6, condensation occurred only in cola/milk mixtures containing 30%–40% cola, but not in those with a lower or higher cola content. This is a phase behavior that is typical of RC, in which a system condenses only at moderate concentrations of multivalent ions or polyelectrolytes (Kumar et al., 2019). Conversely, at neutral pH (pH 7.0–7.4), no condensation was observed at any of the tested concentration of cola. We then focused on casein, which constitutes 2.6% of milk and 79.5% of milk proteins. Casein has a positive charge at pH 3.2–3.6 and a negative charge at pH 7.0–7.4 because its p*I* is 4.6. We observed RC in the cola/casein mixture, similar to that observed in the cola/milk system. The quantification of the concentration of casein in the supernatant revealed that this protein was present in the condensate. Also, adding 100 mM NaCl induced casein aggregation at low and high concentrations of cola, indicating that electrostatic repulsion stabilizes casein. Previous studies have reported the RC of negatively charged proteins in the presence of multivalent cations (Kumar et al., 2019); (Fries et al., 2017). Because casein has a positive charge at pH 3, the multivalent anions present in the cola may have induced the RC of casein. Therefore, we focused on polyP, which is a multivalent polyanion that is included in cola as a food additive (Ido & Komine, 2014). The charge numbers of tetraP, triP, and diP at pH 3.4, as determined by acid titration, were −4.0, −2.9, and −1.9, respectively (Figure S1). As expected, RC occurred in the tetraP/casein mixed solution. Furthermore, the zeta potential changed from positive to negative as the concentration of tetraP increased, indicating the occurrence of a charge inversion, which is a phenomenon that is typical of RC (Zhang et al., 2014); (Lenton et al., 2021). Similarly, a system composed of positively charged lysozyme and triP, which has a charge number of −4.8 at pH 9, underwent RC associated with a charge inversion (Bye & Curtis, 2019).

PolyP is a negatively charged linear polymer comprising three to several thousand inorganic phosphate units. PolyP has been highly conserved in living organisms since the prebiotic era and has been identified in every living organism tested to date (Rao, Gómez-García, & Kornberg, 2009); (Kornberg, 1995). At an acidic pH, polyP interacts with positively charged proteins via electrostatic interactions, thus promoting protein aggregation and amyloid formation (Zhang et al., 2019); (Sasahara, Yamaguchi, So, & Goto, 2019). Depending on chain length, PolyP also induces the LLPS of positively charged green fluorescent protein (+36GFP); (Wang et al., 2020). Interestingly, polyP induces an RC behavior in biomolecules in a pure system. PolyP induces the RC of histatin 5 and lysozyme because of their high density of negative charges (Lenton et al., 2021); (Bye & Curtis, 2019). In this case, arginine interacts more strongly with polyP than does lysine, although they have the same positive charge (Lenton et al., 2021). At moderate concentrations, triphosphate (triP) forms gels with positively charged polyarylamines via electrostatic interactions, whereas triP at a higher concentration dissolves the gels by reversing the surface charge of the triP/polyarylamine complex (Huang, Lawrence, & Lapitsky, 2014). These findings support our observation that positively charged casein interacts with anionic polyP via an electrostatic interaction, eventually inducing RC even in a solution containing contaminants.

Because we observed that polyP was important for the RC of casein in a pure system, polyP was likely to be the main factor causing the RC of the cola/milk mixture. Cola contains approximately 0.20 mg/ml phosphorus (Suntory Holdings Limited, 2023), which corresponds to 6.5 mM phosphate units and is of the same order of magnitude as the concentrations used in the present study. Although cola contains orthophosphate as an acidulant, the addition of 300–1500 ppm polyP (4–19 mM phosphate units) is preferred for maintaining the carbon dioxide bubbles during the production of cola (Ido & Komine, 2014), Cola also contains small amounts of monovalent or divalent ions, such as 0.02 mg/ml sodium, 0.02 mg/ml calcium, and 0.01 mg/ml magnesium (Ministry of Education, Culture, Sports, Science and Technology, 2015); however, in the range of several millimolar, these monovalent or divalent cations do not induce the RC of BSA (Kumar et al., 2019) and silica nanoparticles (Kumar, Yadav, Abbas, Aswal, & Kohlbrecher, 2017). In the case of polyP, triP induces RC of positively charged lysozyme and histatin 5, whereas diP does not (Lenton et al., 2021). Based on the patent of cola, it does not appear to contain trivalent or more multivalent ions except for polyP (Ido & Komine, 2014). Caramel food coloring, which is also a major component of cola, has a negative charge at pH 3.2–3.6 because its p*I* is below 2.5 (Royle & Radcliffe, 1999). It may condense with positively charged casein, resulting in a supernatant with a clear appearance. However, the RC behavior of the tetraP/casein mixed solution was very similar to that of the cola/casein and cola/milk systems. Thus, we concluded that the RC observed in this cola/milk system was induced by an interaction between polyP and casein.

**Fig. 6:**
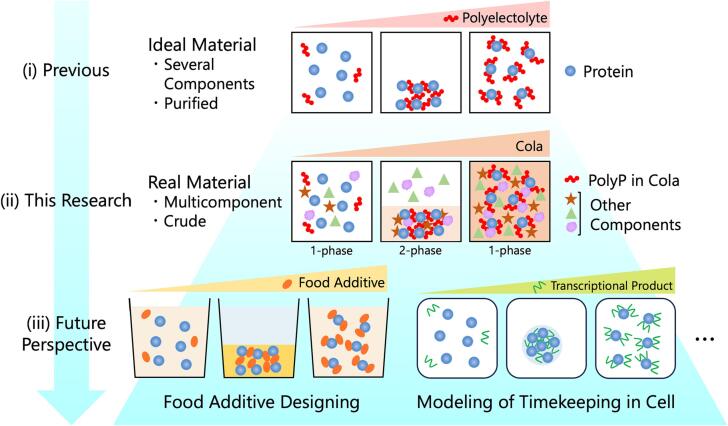
Illustration of RC from previous study to the future. (i) RC has been characterized in the field of physical chemistry using ideal material. (ii) Schematic model of RC of the cola/milk mixture at a pH value below the p*I* of casein revealed in this research. (Left) In the presence of low cola concentrations, the positively charged casein in milk is soluble because of electrostatic repulsion. (Middle) At moderate cola concentrations, the positively charged casein in milk is crosslinked by the anionic multivalent polyP in cola because of charge neutralization. (Right) In the presence of high cola concentrations, the excess of polyP in the cola increases the repulsion between casein molecules because of the charge inversion of casein. (iii) Introducing the concept of RC to real materials would upgrade food additive designing strategy or contribute to modeling of biological phenomenon such as timekeeping in cell.

The polyP-induced RC of casein can be explained by the following mechanism. At an acidic pH below its p*I*, casein has positive charges, and polyP has negative charges. Thus, positively charged casein is crosslinked by anionic multivalent polyP, as observed in the PPC (Kurinomaru & Shiraki, 2017; Matsuda et al., 2018) or the co-aggregation of two types of oppositely charged proteins (Iwashita, Handa, & Shiraki, 2018); (Iwashita, Handa, & Shiraki, 2019). Furthermore, the length of the polyP chain had an effect, as casein condensed with 0.2 mM tetraP and 1 mM triP at pH 2.6, whereas it did not condense with diP. Moreover, 2 mM tetraP solubilized casein. These results indicated the importance of multivalent interactions between casein and polyP for RC. When increasing cola concentrations at a pH below the p*I* of a protein, the system undergoes transitions between the states depicted in Fig. 6 (ii), as follows: (left) a soluble one-phase state, in which casein molecules have repulsive positive charges; (middle) a two-phase state with condensation, in which positively charged casein is crosslinked by anionic multivalent polyP because of charge neutralization; and (right) a soluble one-phase state, in which an excessive amount of polyP increases the repulsion between casein molecules because of a charge inversion in casein. The formation of soluble PPC occurs via a similar mechanism, i.e., aggregative PPC is formed in the presence of low concentrations of polyelectrolytes and redissolves in the presence of high concentrations of polyelectrolytes (Kurinomaru & Shiraki, 2017; Matsuda et al., 2018). The RC of our cola/milk system can be explained by a simple reentrant mechanism, even though the system included many contaminants; therefore, this system is a representative model of RC in complex systems, such as the cytosol.

Theoretically, multicomponent processes, such as food production, would involve RC. In fact, some examples of this contention have been reported. During cheese production, for example, the concentration of diP has an optical value for strongest gel formation with 5.1% milk; i.e., both higher or lower concentrations of diP weaken the gel strength. This process exhibits similarities with RC in that condensation and redispersion events occur concomitant with the increase in one component. Although cheese has been produced for a very long time, this similarity was overlooked, and there are no reports of the occurrence of RC in a non-pure system. The present cola/milk system clearly demonstrated that RC occurred experimentally in a real multicomponent system. The reconsideration of the food production process from the viewpoint of RC management would upgrade the industrial production of processed foods. Moreover, the utilization of this inexpensive model warrants a deep investigation of how and when RC occurs at the industrial scale and in industrial conditions. Our findings provide a bridge between the concepts of RC and LLPS and the food industry.

Not only for food materials, condensed biomolecules, called “complex coacervates” or “condensates,” play important roles in biological processes (De Kruif et al., 2004; Shin & Brangwynne, 2017; Banani et al., 2017). In living cells, such condensed states are termed “membraneless organelles” (MLOs). The components of MLOs can be exchanged with those in the surrounding compartments and are grouped (Banani et al., 2017). Typically, they consist of charged biomolecules, such as proteins, nucleic acids, and polyP (Wang et al., 2020; Brangwynne et al., 2009; Boeynaems et al., 2019).Although the mechanism of MLO formation has been well studied, that underlying MLO dissolution remains poorly understood. RC is one of the candidate mechanisms to explain both the formation and dissolution of MLOs. Some combinations of charged biomolecules, such as RNA/RNA-binding proteins and RNA/positively charged peptides, exhibit an RC behavior because of their high charge density (Banerjee et al., 2017). Molar or submolar concentrations of monovalent or divalent salts can induce the RC of intrinsically disordered proteins, such as FUS, LAF1, and γd-crystallin (Krainer et al., 2021). Recent studies have suggested that RC contributes to biochemical timekeeping by generating condensates that form and dissolve as the concentration of transcription products monotonically increases (Portz & Shorter, 2021). However, the induction of RC has not been achieved in real cells, because it would be difficult to cause an increase in a single component without triggering disturbances. Therefore, to understand the mechanisms underlying RC in complex systems, such as the cytosol, it is important to construct an artificial model that includes multiple complex contaminants.

## 5 Conclusion

In this study, the cola/milk system exhibited RC in the mixture. This RC was understood as involving two main components, polyP in cola and casein in milk, and was explained by the electrostatic shielding and overcharging of casein by polyP. This was the first demonstration of the occurrence of RC in a nonpure system. Our findings not only established a liter-scale and inexpensive model of RC in a multicomponent system, but also strongly supported previous reports of the condensate formation/deformation together with component concentration in contaminated environments, such as cheese materials, as the occurrence of RC. This cola/milk system suggests the transition of the concept of RC in nonpure systems from cell biology to industrial food production.

## CRediT authorship contribution statement

**Tomohiro Furuki:** Conceptualization, Investigation, Writing – original draft. **Tomohiro Nobeyama:** Supervision, Writing – review & editing. **Shunji Suetaka:** Investigation. **Ryokei Matsui:** Investigation. **Tatsuhiko Fukuoka:** Supervision. **Munehito Arai:** Supervision, Writing – review & editing. **Kentaro Shiraki:** Conceptualization, Funding acquisition, Supervision, Writing – review & editing.

## Declaration of competing interest

The authors declare that they have no known competing financial interests or personal relationships that could have appeared to influence the work reported in this paper.

## Data Availability

Data will be made available on request.
