# Supplementary material for: Reentrant condensation of a multicomponent cola/milk system induced by polyphosphate
Source: Food Chem X. 2024 Jan 28;21:101165. doi: 10.1016/j.fochx.2024.101165 (PMC10847600; doi:10.1016/j.fochx.2024.101165)
Supplement: Supplementary data 1 [file mmc1.docx]

**Supplementary Information**


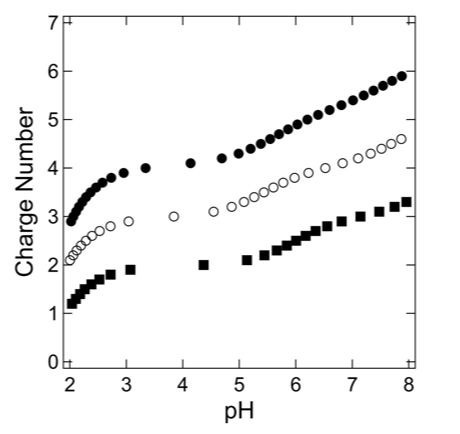


Figure S1. Dependence of the charge number of polyP with varying chain lengths on the pH, as determined by acid titration. Closed circles, tetraP; open circles, triP; closed squares, diP.
